# Os Odontoideum: A Comprehensive Clinical and Surgical Review

**DOI:** 10.7759/cureus.1551

**Published:** 2017-08-08

**Authors:** Fareed Jumah, Saja Alkhdour, Shaden Mansour, Puhan He, Ali Hroub, Nimer Adeeb, Rimal Hanif, Martin M Mortazavi, R. Shane Tubbs, Anil Nanda

**Affiliations:** 1 School of Medicine, An-najah National University Hospital, Nablus, Palestine; 2 Harvard School of Dental Medicine, Harvard University; 3 Department of Neurosurgery, Louisiana State University, Shreveport, LA.; 4 California Institute of Neuroscience, Los Robles Hospital and Medical Center; 5 Neurosurgery, Seattle Science Foundation

**Keywords:** os odontoideum, odontoid, dens, c2, fracture, spine, cervical, review

## Abstract

Os odontoideum (OO) is a rare anomaly of the odontoid process first described by Giacomini in 1886. There is considerable debate about the origin of this anomaly, whether congenital or acquired, though a growing body of evidence favors the latter.

Using PubMed, we reviewed the literature on OO with regards to its etiology, clinical presentations, diagnostic modalities, and management. Manuscripts cited in reviews were also searched manually.

Because the medical literature on this condition is limited, our understanding of the natural history and management of OO is still vague. The management guidelines for asymptomatic OO are preliminary. Therefore, we need more large-center studies to investigate this condition further.

## Introduction and background

**Figure 1:**
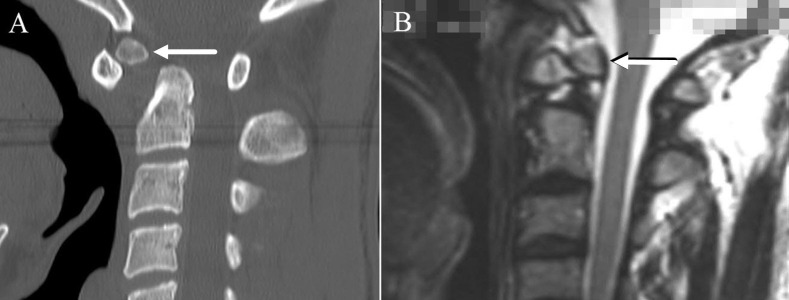
Spine imaging showing the two anatomic subtypes of os odontoideum: dystopic (Panel A) and orthotopic (Panel B)

Os odontoideum (OO) is a congenital anomaly of the second cervical vertebra (axis), defined as a smooth, independent ossicle of variable size and shape separated from the base of a shortened odontoid process by an obvious gap, with no osseous connection to the body of C2 [1]. OO can be classified into two anatomical types, orthotopic and dystopic [2]. (Figure 1) An orthotropic OO lies in the normal position on the odontoid process, moving with the atlas anterior arch, while the dystopic morphology describes an ossicle fused to the basion.

Since the first description by Giacomini in 1886, whether the etiology of OO is congenital or traumatic has remained a subject of controversy. However, regardless of its etiology, OO remains a major compromise to atlanto-axial stability, even under physiological loads in some individuals, placing the spinal cord and the vertebral artery at a significant risk of injury. Moreover, the spectrum of clinical presentations is striking, ranging from mild neurological symptoms to paralysis. 

## Review

Embryology

Defining the anatomy and embryology of the odontoid is crucial for understanding the etiology of OO and for reducing the rates of false-positive diagnoses, especially in the pediatric population. Early in development, the fourth occipital sclerotome forms the apex of the odontoid, which is called the ossiculum terminal or the apical odontoid epiphysis [3], while the first and second cervical sclerotomes contribute to the odontoid and axis bodies, respectively [4-5]. After birth, the odontoid has an epiphyseal growth plate separating the first and second cervical sclerotomes known as the neurocentral synchondrosis, which lies below the level of the superior articular facets of the axis and is usually visible in children younger than three or four years but disappears by eight years of age [6]. The odontoid has a different blood supply from the rest of the spine. The neurocentral synchondrosis precludes a rostral vascular supply by the anterior and posterior branches of the vertebral artery. As a result, the odontoid process depends significantly on a terminal descending supply superiorly, called the apical arcade. This relative deficiency of the odontoid blood supply places it at a significant risk of ischemia and necrosis. The blood supply of the odontoid process comes from two sources and is different from the rest of the spine. The vertebral artery gives off posterior ascending arteries at the level of C3, which supply deep penetrating branches as they pass anterior and posterior to the bodies of the axis and the odontoid process, eventually anastomosing with the apical arcade. Furthermore, the anterior ascending arteries and the apical arcade anastomose with branches from the carotid arteries through the base of the skull and the alar ligaments. This arterial apparatus is crucial early in life since no vessels pass through the transient epiphyseal plate between the odontoid process and the axis. More importantly, the relatively fixed position of the dens as the atlas rotates prevents sufficient vascularization by the anterior and posterior branches of the vertebral arteries. Consequently, the odontoid process depends significantly on a terminal descending supply superiorly (the apical arcade). This relative deficiency of the odontoid blood supply renders it particularly vulnerable to ischemia and necrosis, especially in traumatic events. Moreover, the blood supply of the odontoid process may be unstable because the blood vessels traverse closely alongside the odontoid process and, hence, can be easily obstructed. Such an obstruction leads to ischemia that may contribute to poor fracture healing and callus formation.

Etiology

The etiology of OO remains controversial, although a growing body of evidence favors the traumatic over the congenital hypothesis. According to this hypothesis, OO results from a failure of the dens to fuse with the body of the axis during embryonic development [2] or of the secondary ossification center at the apex of the dens to fuse with its main part [2]. Another possible explanation allowed by this hypothesis is the failure of proper caudal migration of the dens during development [7]. This condition has been described in identical twins [7] and in families, suggesting an autosomal dominant pattern [8]. The congenital etiology is further supported by the association of OO with many congenital syndromes and malformations such as bipartite atlas [9], Morquio’s disease [10], the Klippel-Feil syndrome [11], multiple epiphyseal dysplasia [10], achondroplasia [12], the Larson syndrome [13], the Wolcott-Rallison syndrome [14], and chondrodystrophia calcificans [15]. Authors supporting the congenital hypothesis have proposed that trauma per se could not lead to OO formation. Instead, a traumatic event could increase instability due to soft tissue injury, consequently unmasking a pre-existing OO.

The post-traumatic or acquired hypothesis originated mainly from the work of Fielding and Griffin [16], who proposed that OO forms after an unrecognized fracture to the odontoid with the subsequent contraction of the apical and alar ligaments, the distraction of the fractured fragment, and the severing of blood supply, leading to the formation of an ossicle, the OO. Several other studies have supported the post-traumatic etiology [17-19,40]. Proponents of this hypothesis argue that OO is most commonly located at the base of the dens and not at the synchondrosis, where a congenital fusion failure would be expected to occur. In addition, Verska and Anderson [20] presented a case report of a post-traumatic OO in one identical twin, the other twin having a completely normal cervical radiograph with no history of trauma, which argues against a congenital etiology. Case report investigations of children with OO indicate a vascular etiology, such as post-traumatic avascular necrosis [21], or post-traumatic blood supply blockage to the proximal odontoid with osseous absorption, where the ossiculum terminale continues to receive a normal blood supply, forming the OO [22]. Nevertheless, a combination of both etiologies is supported by the argument that deficient ossification of the odontoid and hyperlaxity of ligaments in the previously mentioned congenital syndromes predispose individuals to traumatic OO [23]. However, regardless of the etiology, diagnosis and management remain the same [24].

Clinical Presentation and Complications

Since the first description of OO, researchers have enriched the literature with numerous case reports and series related to the condition. The exact incidence and prevalence remain unclear because of the rarity of this anomaly and its silent course in many individuals [25]. However, while looking for anatomical variations of the odontoid process, Perdikakis et al. found that 0.7 percent of patients examined for probable cervical spine pathology had OO, and Sankar et al. detected it in 3.1 percent of children with abnormal cervical radiographs [26-27]. Although the etiology, natural history, and need for surgery remain controversial, clinical presentation among patients with OO can be classified into four main categories: incidental finding in asymptomatic patients, local symptoms, cervical myelopathic symptoms and signs, and symptoms related to vertebrobasilar ischemia [28]. This wide range of manifestations can be attributed to multiple factors, such as the slow progression of atlanto-axial instability and irritation caused by an OO [16], the anatomical type of OO (dystopic vs. orthotopic) [29], and the radiographic morphology of the atlanto-axial joint, the round type being associated with more severe myelopathic manifestations than either the cone or blunt-tooth types [30].

The onset of symptoms is noticeably related to traumatic events, including minor ones, and patients are frequently diagnosed during adolescence and early adulthood [31-32]. Neck pain and stiffness, shoulder pain, torticollis, and occipital headaches are the most common local symptoms in patients with OO [31-32]. Several atypical symptoms have also been reported. For example, Zussman et al. presented a case of chronic posterior thoracic pain refractory to symptomatic treatment, which was later diagnosed with cervical instability secondary to OO [33]. However, because of the high prevalence of symptoms such as headaches and neck pain in the general population, their association with OO is difficult to establish.

Patients with myelopathy can present with transient or progressive symptoms related to the site and degree of compression [25,32]. The literature documents a wide range of such symptoms, including weakness, numbness, and paresthesias [2], the central cord syndrome, the Brown-Séquard syndrome [34], Lhermitte’s phenomenon [35], the central hypoventilation syndrome (Ondine’s curse) [29], and cardiorespiratory arrest and sudden death [36]. One case report described the association of OO with the ossification of the posterior atlanto-axial membrane in an adult patient with severe myelopathy and assumed that the ossification, which is a rare cause of myelopathy, was a consequence of chronic mechanical stress on the ligament [37].

OO has also been implicated in the development of cerebellar infarction [38]. Three-dimensional (3D) computed tomographic (CT) angiography showed that atlanto-axial dislocation resulted in the irregular narrowing of the vertebral artery, so the endothelium there is a likely focus for embolus formation [39].

Differential Diagnosis of OS

Based on history and physical exam, the clinical manifestations of OO may resemble a myriad of other conditions, most notably degenerative disc disease of the cervical spine, cervical spondylosis, and Grade II mechanical neck pain, or atlantoaxial subluxation (e.g., due to rheumatoid arthritis).

On the other hand, the radiographic differential diagnosis of OO is limited. The main diagnosis to consider is an acute fracture of the odontoid process. OO can be distinguished by the smooth surface of the ossicle and the underlying body peg of C2, the absence of a recent history of trauma, and the possible sclerosis and hypertrophy of the anterior tubercle of the atlas. A persistent ossiculum terminale is another differential diagnosis that may be confused with OO. It is caused by the nonunion of the apex at the secondary ossification center. However, it is rarely associated with C1-C2 instability and, consequently, does not require surgical correction.

Radiological Findings and Imaging Modalities in the Diagnosis and Assessment of OO

Broadly speaking, OO can be clearly visualized using plain radiographs with the open mouth, anteroposterior, and lateral views. In addition, plain dynamic lateral radiographs (performed in flexion and extension) can further evaluate atlanto-axial instability. Nevertheless, the sensitivity and specificity of these imaging modalities have not been studied [25]. On the other hand, CT scans, CT scans with angiography, and magnetic resonance imaging (MRI) scans are important for a better illustration of osseous abnormalities, the arrangement of vertebral arteries, and spinal cord compression and pathology, respectively. Such illustration is mandatory for identifying the exact causes of the patients' symptoms and planning for surgery [25]. Furthermore, Hughes et al. recommended the use of kinematic MRI in diagnosing OO, given the advantage of directly visualizing the motions of joint components and the surrounding soft tissues [41]. However, an initial examination of patients with myelopathy using a conventional MRI scan can occasionally lead to a misdiagnosis of chronic cervical spine instability secondary to OO as an intramedullary spinal cord tumor [42].

In attempts to correlate symptomatic status with accurate parameters, several indicators for assessing instability have been suggested. The most commonly used ones are the direction of atlanto-axial instability, whether anterior (most common), posterior, or multidirectional [43], the space available for the spinal cord (using 13 mm as a cut-off) [44], and the instability index ( more than 40 percent being significant) [45]. Nonetheless, many researchers have concluded that such parameters cannot reflect the true degree of instability [46]. Moreover, using MRI scans, Chang et al. suggested that myelopathy in patients with OO is a result of retrodental cystic and fibrocartilaginous masses rather than atlanto-axial instability [47].

This considerable concern about suitable parameters is mainly attributable to the need to set clear guidelines for managing patients with OO, especially those who are asymptomatic.

Cadaveric Findings

In the first study of its kind, Sardi et al. report the cadaveric findings of a 72-year-old male with OO but no history of craniocervical instability. The cause of death was unknown. On gross examination, the specimen did not display any arthritic degeneration. The proximal portion of the odontoid process was observed as a clear continuation of the axis body, measuring 12.96 x 11.16 x 11.93 mm (L x D x W). However, the distal fragment measured 10.03 x 6.06 x 4.03 mm (L x D x W) and was separated from the rest of the odontoid process by a small gap. The OO was orthotopic, quite mobile, and in contact with the odontoid process with evidence of remodeling, but no articular cartilage was observed. Interestingly, however, the CT measurements of the OO were almost half as large (5.15 x 5.39 x 3.84 mm (L x W x D)).

Management of OO

The management guidelines for OO remain vague because the evidence in the literature is limited, especially since the condition is rare and its natural history poorly understood. There is general agreement that patients with symptomatic OO (e.g. cervical myelopathy) should be treated surgically. However, debate still continues regarding the treatment of asymptomatic OO.

Dai et al. noted that five cases of asymptomatic OO that were managed conservatively remained stable at follow-up more than one year later [48]. Others have found no difference in outcome between the conservative and surgical treatment of patients with asymptomatic OO [32,49]. Therefore, most authors believe a conservative approach is adequate if OO is asymptomatic, while clinical and radiological follow-ups are used to monitor for radiographic instability or significant symptoms [25,43,46,50]. However, a subset of asymptomatic patients is thought to be at risk for deterioration, so these patients should be considered for prophylactic spinal fusion. For example, Spierings and Braakman [32] found that asymptomatic OO patients with a minimal sagittal diameter of less than 13 mm had the greatest risk of spinal cord injury. Other asymptomatic patients who are potential candidates for surgical intervention are young, have favorable anatomy, and show radiographic evidence of atlanto-axial instability on flexion extension X-rays [43].

On the other hand, because evidence concerning the long-term natural course of untreated OO is limited, some authors [31] believe that all asymptomatic patients should undergo C1-C2 fusion to avert neurological complications, even those with a ‘stable’ OO. This can be better appreciated when we consider reports in the literature on sudden death [36,48], significant neurological complications [10] following minor injuries in previously undiagnosed OO, and patients who suffer late neurological deterioration [31]. Moreover, these patients are at increased risk of neurological injury during the flexion and extension of the neck during intubation and patient positioning [47]. For those reasons, Class III medical evidence in the literature suggests that prophylactic C1-C2 fusion is meritorious in such cases [50].

It is worth mentioning that surgeons have attempted to define radiographic risk factors that predict a high risk of spinal cord injury in asymptomatic patients. Atlanto-axial instability has been defined as more than 3 mm of motion at C1-C2 on flexion-extension films [2]. However, neither cervical flexion-extension X-rays nor the degree of atlanto-axial subluxation on static imaging (CT, MRI, and plain films) has been shown to correlate with neurological status [32]. Nevertheless, some authors found that a sagittal spinal canal diameter less than 13 mm was strongly associated with myelopathy [32], while patients with transient or progressive myelopathy had an instability index of more than 40 percent or a sagittal plane rotation more than 20̊ larger than in patients without neurological symptoms [44]. The “round” type of OO morphology correlates more strongly with myelopathy [30]. Age is another important factor in managing asymptomatic OO. Children, for example, are more prone to falls from day-to-day activities, so they are more likely to need protection against the inherently unstable OO [43]. Moreover, although daily activities can be modified to reduce the risk of injury (e.g., contact sports), incidental injuries and road traffic accidents remain a risk factor shared among all age groups.

Regarding the management of symptomatic OO, the literature still lacks clear, high-quality Class I or II evidence, and current management guidelines all come from Class III evidence (case reports and case series). The most common technique is posterior C1-C2 fixation and fusion, although other successful approaches have been used [50]. 

## Conclusions

Our knowledge of this rare and poorly understood condition continues to evolve. Physicians should be familiar with OO and its wide array of manifestations and provide appropriate management that can prevent potentially catastrophic consequences. While there is agreement about the decision to operate on symptomatic OO, patients with asymptomatic OO should be approached more carefully. Physicians should educate them about the unpredictable course of their condition and discuss the available treatment options while keeping in mind that surgery does not come without risks. Given the whole view, the latest recommendation regarding the management of asymptomatic patients suggests either regular clinical and radiographic follow-up or posterior internal fixation and fusion of C1-C2. The natural history of OO still remains vague. More studies, such as a multicenter natural history study, are needed to improve our understanding of this condition and to produce high-quality evidence for its management.
